# Supplementary material for: Engineered Campylobacter jejuni Cas9 variant with enhanced activity and broader targeting range
Source: Commun Biol. 2022 Mar 8;5:211. doi: 10.1038/s42003-022-03149-7 (PMC8904486; doi:10.1038/s42003-022-03149-7)
Supplement: Supplementary file 2 — Supplementary Information [file 42003_2022_3149_MOESM2_ESM.pdf]

Supplementary Materials for

Engineered *Campylobacter jejuni* Cas9 variant with enhanced activity and broadar targeting range

Ryoya Nakagawa, Soh Ishiguro, Sae Okazaki, Hideto Mori, Mamoru Tanaka, Hiroyuki Aburatani, Nozomu Yachie, Hiroshi Nishimasu,*, Osamu Nureki,*

*Correspondence to: nisimasu@g.ecc.u-tokyo.ac.jp; nureki@bs.s.u-tokyo.ac.jp

This PDF file includes:

Supplementary Figures 1 to 7
Supplementary Tables 1 to 4

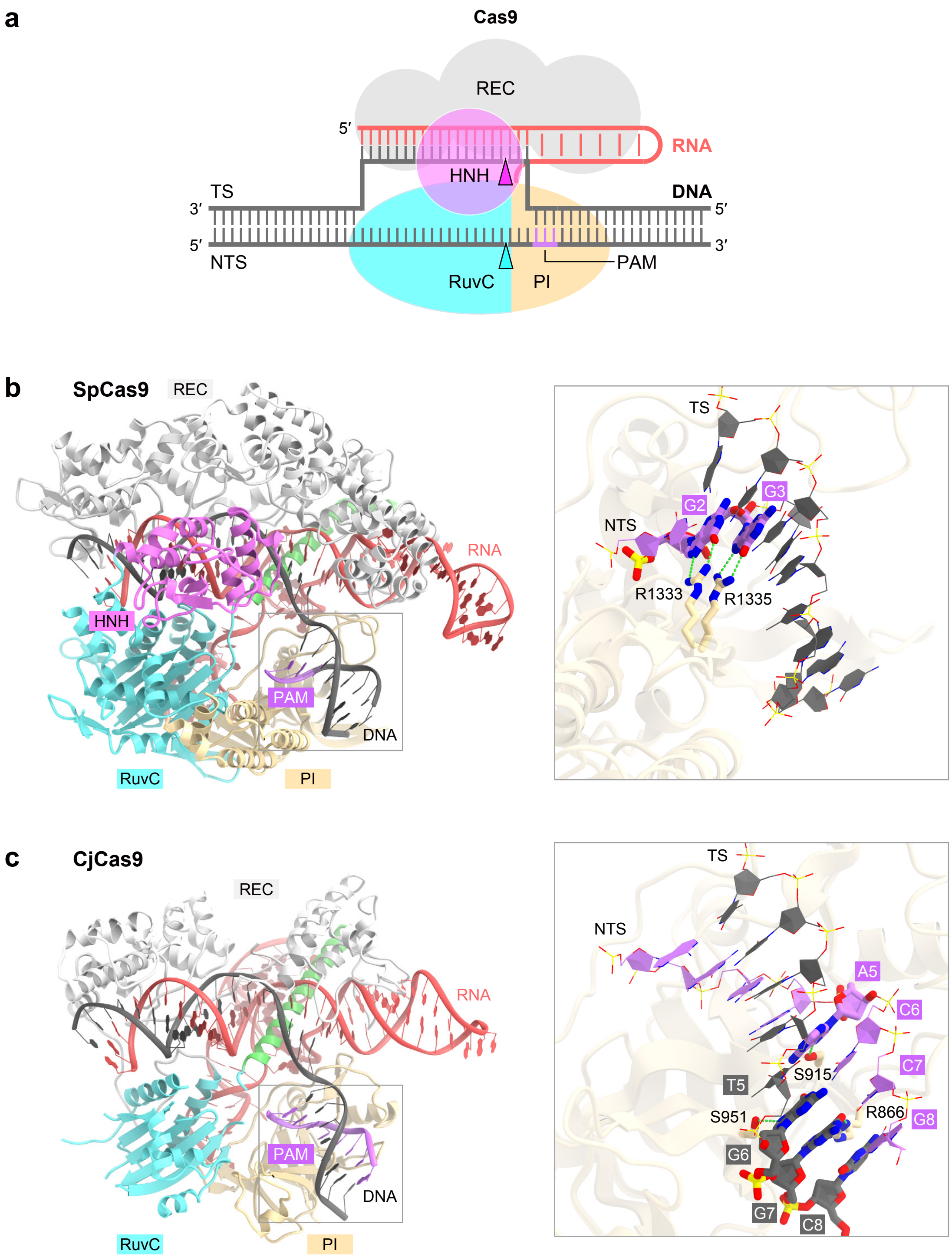

Supplementary Figure 1. RNA-guided DNA cleavage by Cas9
(**a**) Schematic of Cas9-mediated DNA cleavage. PI, PAM-interacting domain; TS, target DNA strand; NTS, non-target DNA strand.
(**b, c**) Crystal structures of SpCas9 (PDB 4UN3) (**b**) and CjCas9 (PDB 5X2G) (**c**) in complex with their guide RNAs and target DNAs. PAM recognitions by their PI domains are shown in insets. The HNH domain of CjCas9 was truncated for the crystallization.

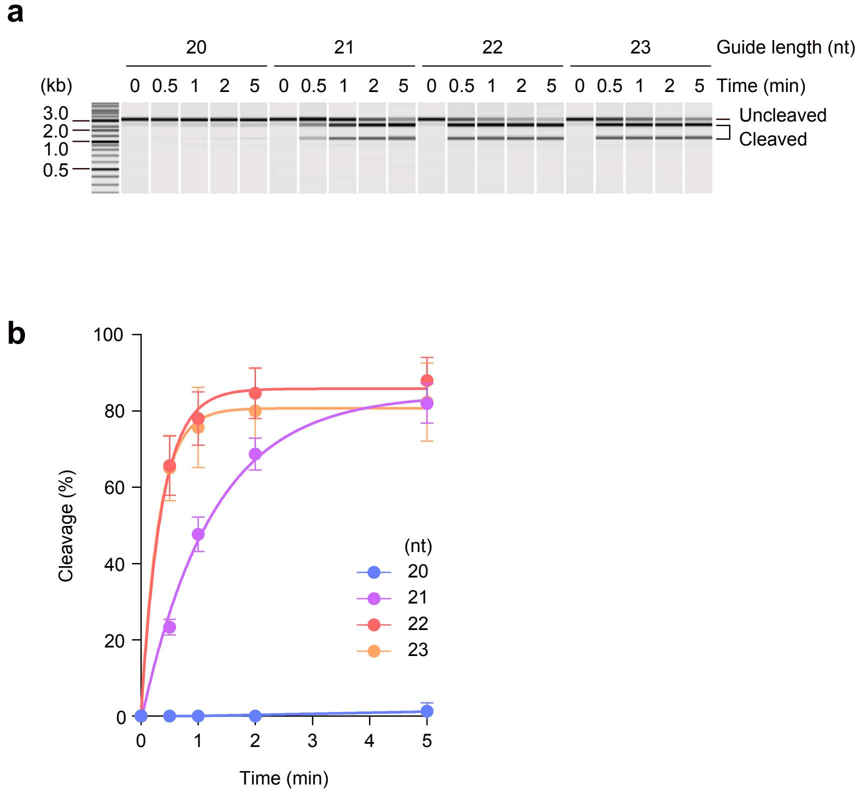

Supplementary Figure 2. Effects of the guide lengths on the CjCas9-mediated DNA cleavage.

(**a**) *In vitro* DNA cleavage activities of CjCas9 with the 20–23-nt guide sgRNAs. The linearized plasmid target bearing the T3AACAC PAM was incubated with the CjCas9–sgRNA complex at 37°C for 0.5, 1, 2, and 5 min. The cleavage products were then analyzed by a MultiNA microchip electrophoresis system.

(**b**) Quantification of the DNA cleavage data in (**a**). Data are mean ± s.d. (n = 3).

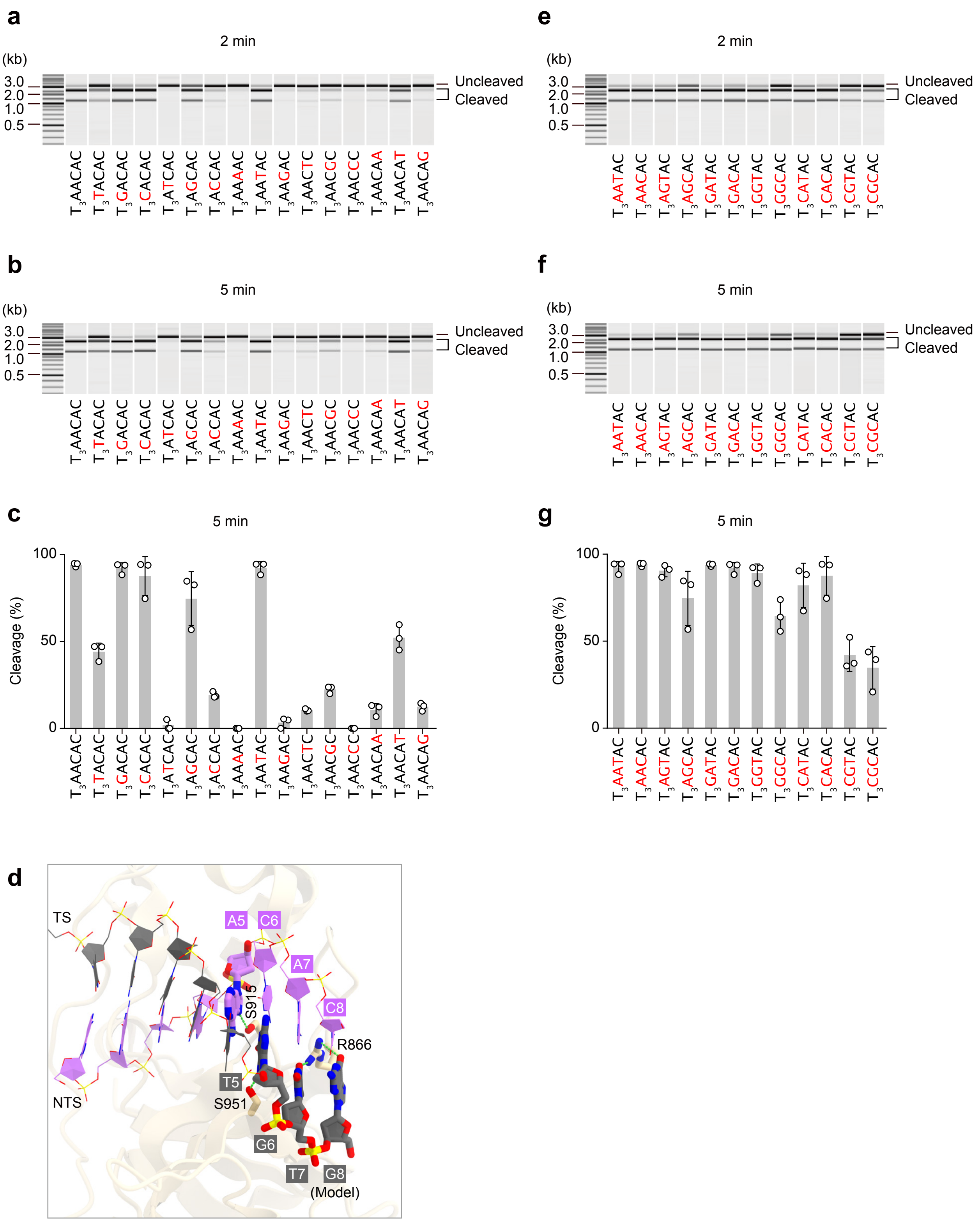

a
2 min
(kb)
3.0
2.0
1.0
0.5
Uncleaved
Cleaved
T3AACAC
T3TACAC
T3GACAC
T3CACAC
T3ATCAC
T3AGCAC
T3ACCAC
T3AAAAC
T3AATAC
T3AAGAC
T3AACTC
T3AACGC
T3AACCC
T3AACAA
T3AACAT
T3AACAG
b
5 min
c
5 min
Cleavage (%)
100
50
0
d
TS
NTS
A5
C6
A7
C8
S915
R866
T5
S951
G6
T7
G8
(Model)
e
2 min
f
5 min
g
5 min
T3AATAC
T3AACAC
T3AGTAC
T3AGCAC
T3GATAC
T3GACAC
T3GGTAC
T3GGCAC
T3CATAC
T3CACAC
T3CGTAC
T3CGCAC

Supplementary Figure 3. *In vitro* cleavage activities of CjCas9.
(**a, b**) *In vitro* DNA cleavage activities of CjCas9 toward 16 PAM targets. The linearized plasmid target bearing the indicated PAM was incubated with the CjCas9–sgRNA (22-nt guide) complex at 37°C for 2 min (**a**) and 5 min (**b**).
(**c**) Quantification of the DNA cleavage data in (**b**). Data are mean ± s.d. (n = 3).
(**d**) Structural explanation of the preference of CjCas9 for the seventh A and the eighth C in the N3VRYAC PAM. The eighth C:G pair in the PAM duplex was modeled into the crystal structure of the CjCas9–guide RNA–target DNA complex (PDB 5X2H, AGAAACAG PAM). Possible hydrogen bonds between Arg866 and the PAM-complementary seventh T and eighth G can explain the preference of CjCas9 for the seventh A and the eighth C in the N_3VRYAC PAM.
(**e, f**) *In vitro* DNA cleavage activities of CjCas9 toward 12 PAM targets. The linearized plasmid target bearing the indicated PAM was incubated with the CjCas9–sgRNA (22-nt guide) complex at 37°C for 2 min (**e**) and 5 min (**f**).
(**g**) Quantification of the DNA cleavage data in (f). Data are mean ± s.d. (n = 3).

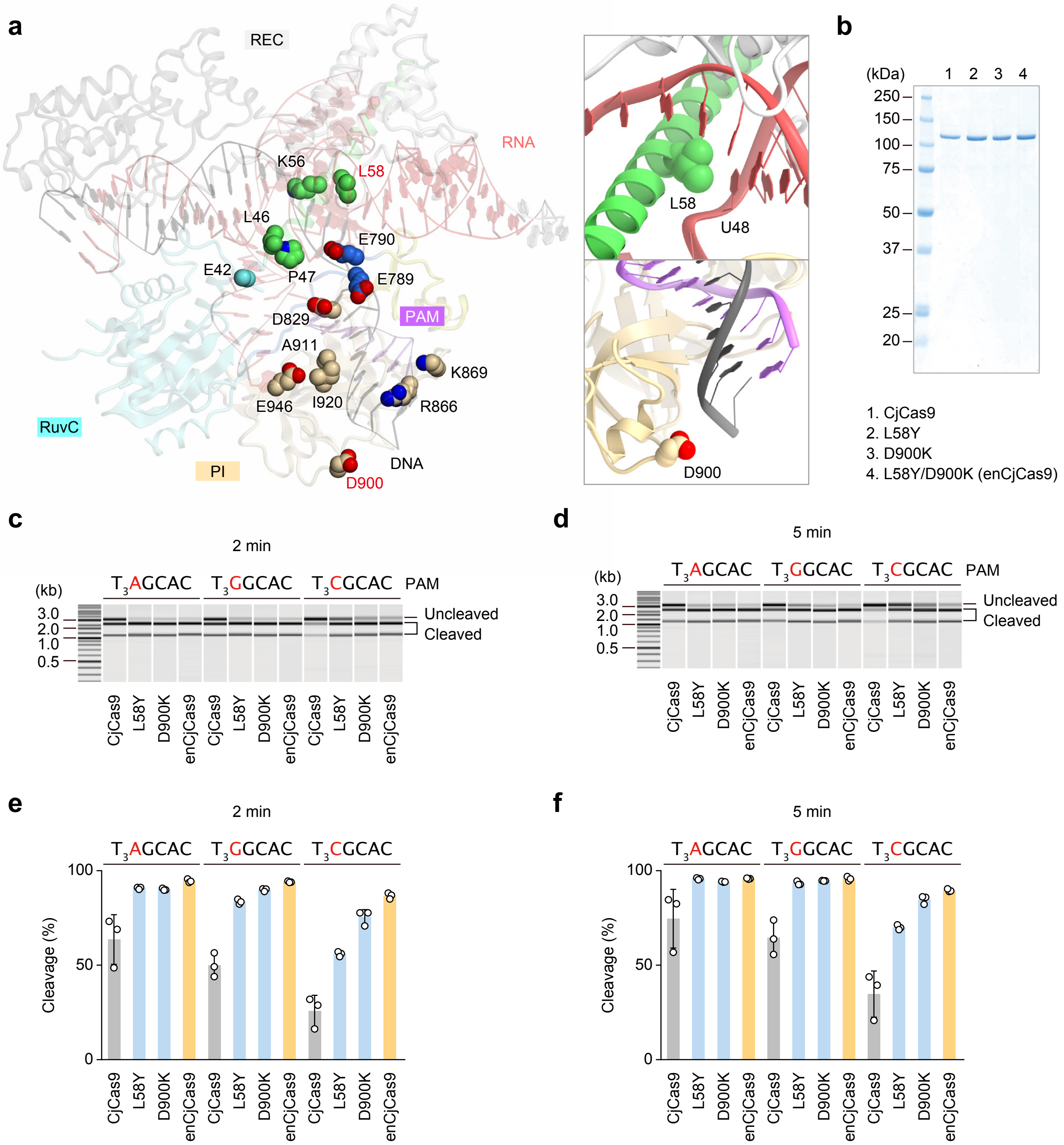

Supplementary Figure 4. Engineering of enCjCas9.

(**a**) Mapping of the 14 mutated residues onto the CjCas9–guide RNA-target DNA complex (PDB 5X2G). Leu58 is located in the bridge helix, suggesting that Tyr58 (L58Y) forms a stacking interaction with U48 of the guide RNA. Asp900 is located in the PI domain, suggesting that Lys900 (D900K) interacts with the phosphate backbone of the PAM duplex. Since mutations, except for L58Y and D900K, did not substantially improve CjCas9-mediated DNA cleavage, we focused on the L58Y and D900K mutations.

(**b**) SDS-PAGE analysis (10–20%) of the wild-type and mutant CjCas9 proteins used for *in vitro* cleavage experiments.

(**c, d**) *In vitro* DNA cleavage activities of the wild-type CjCas9 and the CjCas9 mutants toward sub-optimal PAM targets. The linearized plasmid target bearing the indicated PAM was incubated with the CjCas9–sgRNA (22-nt guide) complex at 37°C for 2 min (**c**) and 5 min (**d**).

(**e, f**) Quantification of the DNA cleavage data in (**c** and **d**). Data are mean ± s.d. (n = 3).

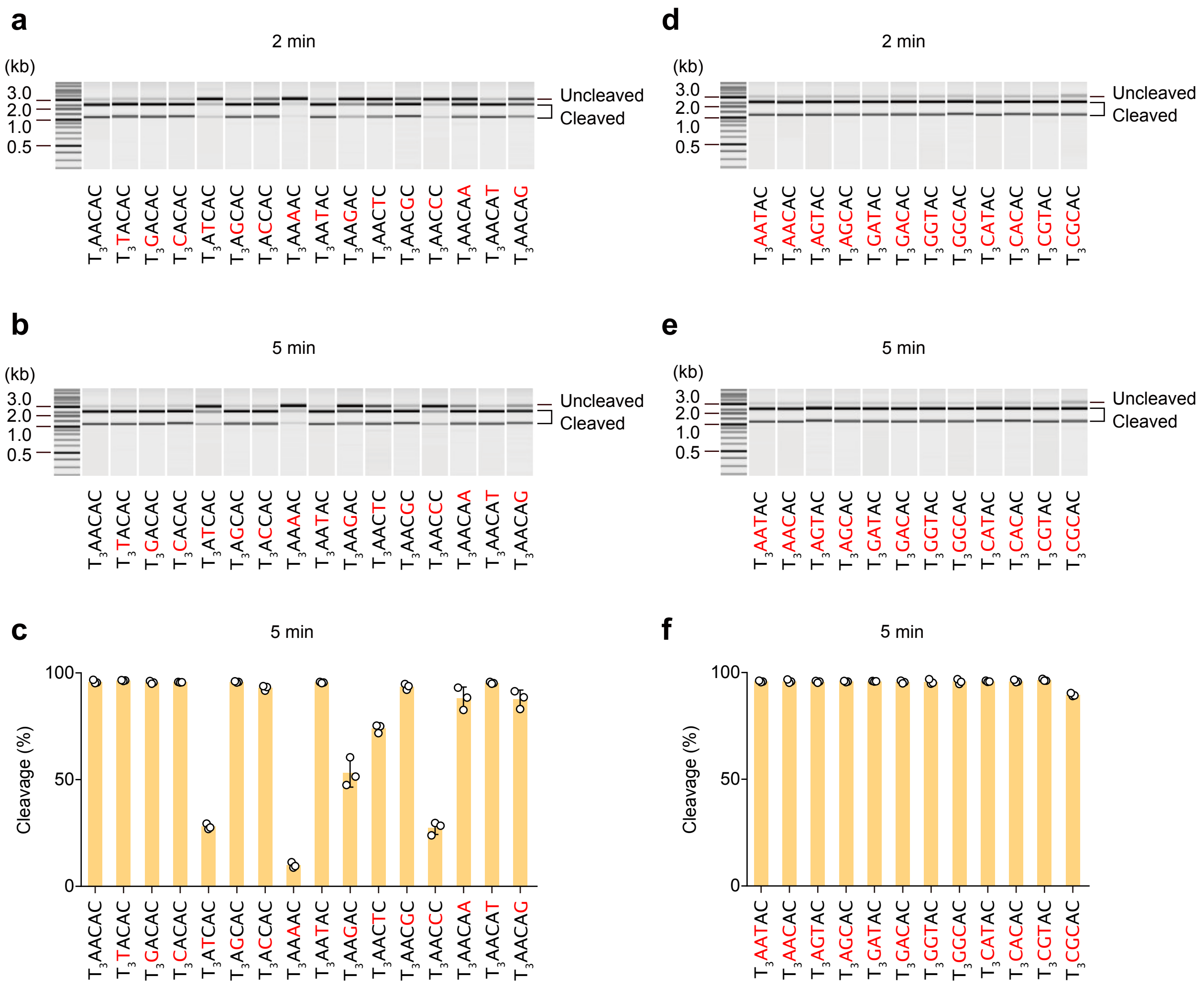

Supplementary Figure 5. *In vitro* cleavage activities of enCjCas9.
(**a, b**) *In vitro* DNA cleavage activities of enCjCas9 toward 16 PAM targets. The linearized plasmid target bearing the indicated PAM was incubated with the CjCas9–sgRNA (22-nt guide) complex at 37°C for 2 min (**a**) and 5 min (**b**).
(**c**) Quantification of the DNA cleavage in (**b**). Data are mean ± s.d. (n = 3).
(**d, e**) *In vitro* DNA cleavage activities of enCjCas9 toward 12 PAM targets. The linearized plasmid target bearing the indicated PAM was incubated with the CjCas9–sgRNA (22-nt guide) complex at 37°C for 2 min (**d**) and 5 min (**e**).
(**f**) Quantification of the DNA cleavage in (**e**). Data are mean ± s.d. (n = 3).

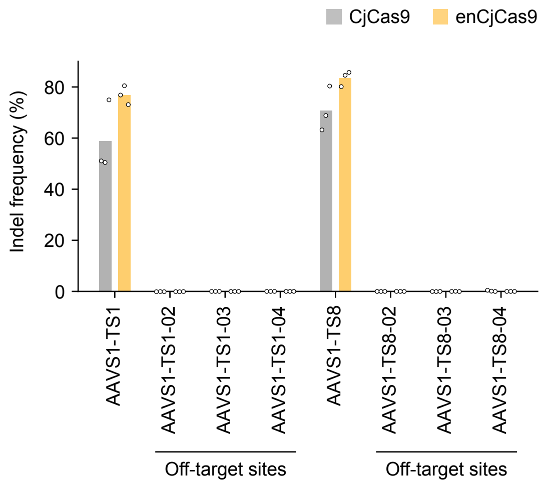

Supplementary Figure 6. Specificities of CjCas9 and enCjCas9.
Indel formation induced by CjCas9 (gray) and enCjCas9 (orange) at the on-target and off-target sites in HEK293Ta cells (n = 3).

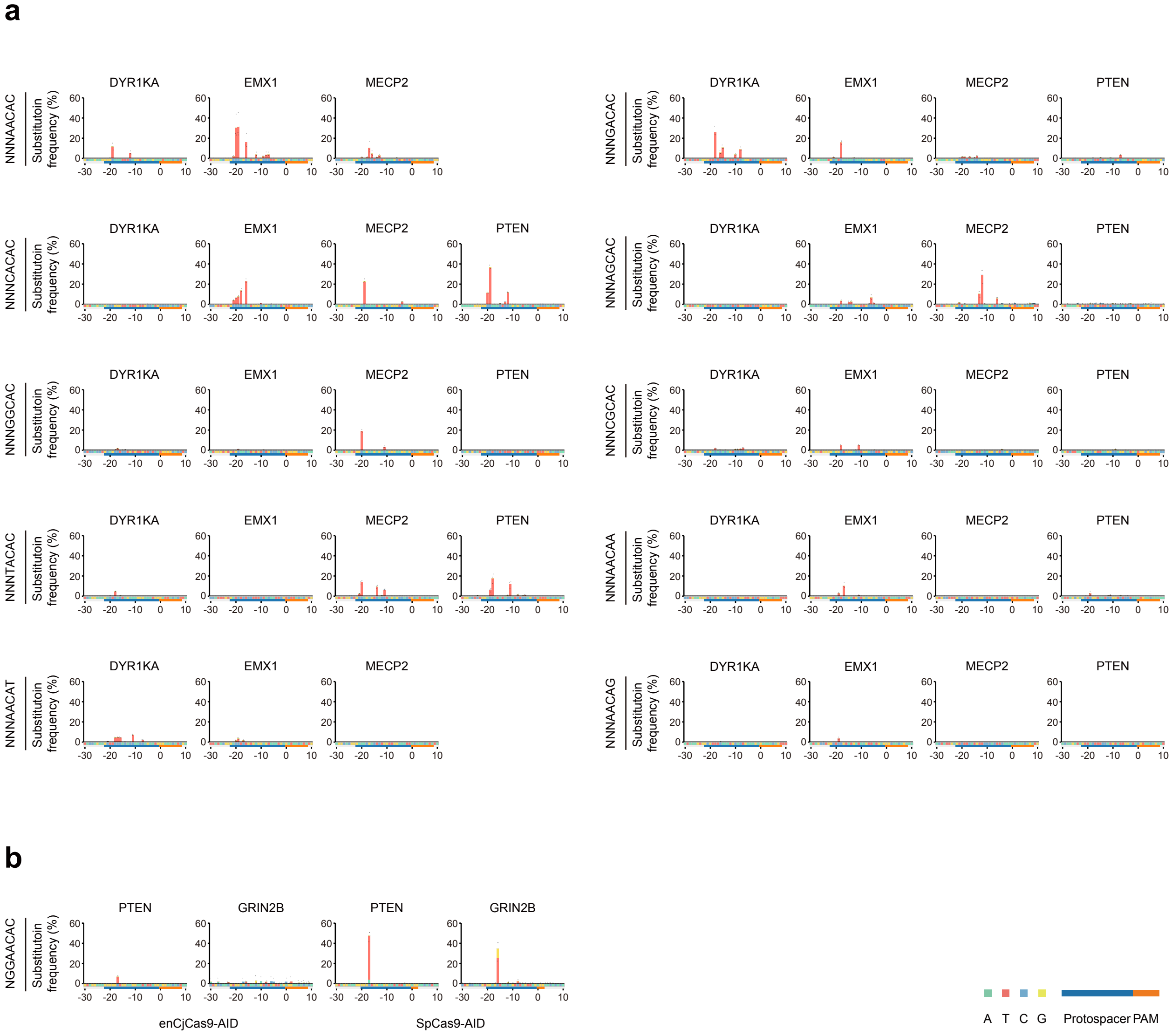

Supplementary Figure 7. Base editing in human cells.
(**a, b**) Base editing spectra of enCjCas9-AID (**a**) and SpCas9-AID (**b**).

Supplementary Table 1. Guide RNA and target DNA sequences used for *in vitro* cleavage experiments.

Name	Sequence
sgRNA-20	GGAAATTAGGTGCGCTTGGCGTTTTAGTCCCTGAAAAGGGACTAAAATAA<br>AGAGTTTGCGGGACTCTGCGGGGTTACAATCCCCTAAAACCGC
sgRNA-21	GGGAAATTAGGTGCGCTTGGCGTTTTAGTCCCTGAAAAGGGACTAAAATA<br>AAGAGTTTGCGGGACTCTGCGGGGTTACAATCCCCTAAAACCGC
sgRNA-22	GGGGAAATTAGGTGCGCTTGGCGTTTTAGTCCCTGAAAAGGGACTAAAAT<br>AAAGAGTTTGCGGGACTCTGCGGGGTTACAATCCCCTAAAACCGC
sgRNA-23	GGGGGAAATTAGGTGCGCTTGGCGTTTTAGTCCCTGAAAAGGGACTAAAA<br>TAAAGAGTTTGCGGGACTCTGCGGGGTTACAATCCCCTAAAACCGC
Target DNA	AGCGCCCAATACGCAAACCGCCTCTCCCCGCGCGTTGGCCGATTCATTAA<br>TGCAGCTGGCACGACAGGTTTCCCGACTGGAAAGCGGGCAGTGAGCGCA<br>ACGCAATTAATGTGAGTTAGCTCACTCATTAGGCACCCCAGGCTTTACAC<br>TTTATGCTTCCGGCTCGTATGTTGTGTGGAATTGTGAGCGGATAACAATTT<br>CACACAGGAAACAGCTATGACCATGATTACGCCAAGCTTGCATGCCTGCA<br>GGTCGACTCTAGAGGATCCCCGGGTACCGAGCTCGAATTCACTGGCCGTC<br>GTTTTACAACGTCGTGACTGGGAAAACCCTGGCGTTACCCAACTTAATCG<br>CCTTGCAGCACATCCCCCTTTCGCCAGCTGGCGTAATAGCGAAGAGGCCC<br>GCACCGATCGCCCTTCCCAACAGTTGCGCAGCCTGAATGGCGAATGGCGC<br>CTGATGCGGTATTTTCTCCTTACGCATCTGTGCGGTATTTCACACCGCATA<br>CGTCAAAGCAACCATAGTACGCGCCCTGTAGCGGCGCATTAAGCGCGGCG<br>GGTGTGGTGGTTACGCGCAGCGTGACCGCTACACTTGCCAGCGCCCTAGC<br>GCCCGCTCCTTTCGCTTTCTTCCCTTCCTTTCTCGCCACGTTCGCCGGCTTT<br>CCCCGTCAAGCTCTAAATCGGGGGCTCCCTTTAGGGTTCCGATTTAGTGCT<br>TTACGGCACCTCGACCCCAAAAAACTTGATTTGGGTGATGGTTCACGTAG<br>TGGGCCATCGCCCTGATAGACGGTTTTTCGCCCTTTGACGTTGGAGTCCAC<br>GTTCTTTAATAGTGGACTCTTGTTCCAAACTGGAACAACACTCAACCCTAT<br>CTCGGGCTATTCTTTTGATTTATAAGGGATTTTGCCGATTTCGGCCTATTG<br>GTTAAAAAATGAGCTGATTTAACAAAAATTTAACGCGAATTTTAACAAAA<br>TATTAACGTTTACAATTTTATGGTGCACTCTCAGTACAATCTGCTCTGATG<br>CCGCATAGTTAAGCCAGCCCCGACACCCGCCAACACCCGCTGACGCGCCC<br>TGACGGGCTTGTCTGCTCCCGGCATCCGCTTACAGACAAGCTGTGACCGT<br>CTCCGGGAGCTGCATGTGTCAGAGGTTTTCACCGTCATCACCGAAACGCG<br>CGAGACGAAAGGGCCTCGTGATACGCCTATTTTTATAGGTTAATGTCATG<br>ATAATAATGGTTTCTTAGACGTCAGGTGGCACTTTTCGGGGAAATGTGCG<br>CGGAACCCCTATTTGTTTATTTTTCTAAATACGGGGGAAATTAGGTGCG<br>CTTGGCTTTAACACGTATCCGCTCATGAGACAATAACCCTGATAAATGCT<br>TCAATAATATTGAAAAAGGAAGAGTATGAGTATTCAACATTTCCGTGTCG<br>CCCTTATTCCCTTTTTTGCGGCATTTTGCCTTCCTGTTTTTGCTCACCCAGA<br>AACGCTGGTGAAAGTAAAAGATGCTGAAGATCAGTTGGGTGCACGAGTG<br>GGTTACATCGAACTGGATCTCAACAGCGGTAAGATCCTTGAGAGTTTTCG<br>CCCCGAAGAACGTTTTCCAATGATGAGCACTTTTAAAGTTCTGCTATGTG<br>GCGCGGTATTATCCCGTATTGACGCCGGGCAAGAGCAACTCGGTCGCCGC<br>ATACACTATTCTCAGAATGACTTGGTTGAGTACTCACCAGTCACAGAAAA<br>GCATCTTACGGATGGCATGACAGTAAGAGAATTATGCAGTGCTGCCATAA<br>CCATGAGTGATAACACTGCGGCCAACTTACTTCTGACAACGATCGGAGGA<br>CCGAAGGAGCTAACCGCTTTTTTGCACAACATGGGGGATCATGTAACTCG<br>CCTTGATCGTTGGGAACCGGAGCTGAATGAAGCCATACCAAACGACGAG<br>CGTGACACCACGATGCCTGTAGCAATGGCAACAACGTTGCGCAAACTATT<br>AACTGGCGAACTACTTACTCTAGCTTCCCGGCAACAATTAATAGACTGGA<br>TGGAGGCGGATAAAGTTGCAGGACCACTTCTGCGCTCGGCCCTTCCGGCT<br>GGCTGGTTTATTGCTGATAAATCTGGAGCCGGTGAGCGTGGGTCTCGCGG

Target DNA	TATCATTGCAGCACTGGGGCCAGATGGTAAGCCCTCCCGTATCGTAGTTA<br>TCTACACGACGGGGAGTCAGGCAACTATGGATGAACGAAATAGACAGAT<br>CGCTGAGATAGGTGCCTCACTGATTAAGCATTGGTAACTGTCAGACCAA<br>GTTTACTCATATATACTTTAGATTGATTTAAAACTTCATTTTTAATTTAAA<br>AGGATCTAGGTGAAGATCCTTTTTGATAATCTCATGACCAAAATCCCTTA<br>ACGTGAGTTTTCGTTCCACTGAGCGTCAGACCCCGTAGAAAAGATCAAA<br>GGATCTTCTTGAGATCCTTTTTTTCTGCGCGTAATCTGCTGCTTGCAAACA<br>AAAAAACCACCGCTACCAGCGGTGGTTTGTTTGCCGGATCAAGAGCTAC<br>CAACTCTTTTTCCGAAGGTAACTGGCTTCAGCAGAGCGCAGATACCAAA<br>TACTGTCCTTCTAGTGTAGCCGTAGTTAGGCCACCACTTCAAGAACTCTG<br>TAGCACCGCCTACATACCTCGCTCTGCTAATCCTGTTACCAGTGGCTGCT<br>GCCAGTGGCGATAAGTCGTGTCTTACCGGGTTGGACTCAAGACGATAGT<br>TACCGGATAAGGCGCAGCGGTCGGGCTGAACGGGGGGTTCGTGCACACA<br>GCCCAGCTTGGAGCGAACGACCTACACCGAACTGAGATACCTACAGCGT<br>GAGCTATGAGAAAGCGCCACGCTTCCCGAAGGGAGAAAGGCGGACAGG<br>TATCCGGTAAGCGGCAGGGTCGGAACAGGAGAGCGCACGAGGGAGCTT<br>CCAGGGGGAAACGCCTGGTATCTTTATAGTCCTGTCGGGTTTCGCCACCT<br>CTGACTTGAGCGTCGATTTTTGTGATGCTCGTCAGGGGGGCGGAGCCTAT<br>GGAAAAACGCCAGCAACGCGGCCTTTTTACGGTTCCTGGCCTTTTGCTGG<br>CCTTTTGCTCACATGTTCTTTCCTGCGTTATCCCCTGATTCTGTGGATAAC<br>CGTATTACCGCCTTTGAGTGAGCTGATACCGCTCGCCGCAGCCGAACGA<br>CCGAGCGCAGCGAGTCAGTGAGCGAGGAAGCGGAA

Supplementary Table 2. Oligonucleotides used in this study.

Oligo	Sequence
sgRNA-T7P-f	GGATCCTAATACGACTCACTATA
sgRNA-20-r	GCGGTTTTAGGGGATTGTAACCCCGCAGAGTCCCGCAAACTCTTTATTTTA GTCCCTTTTCAGGGACTAAAACGCCAAGCGCACCTAATTTCCTATAGTGA GTCGTATTAGGATCC
sgRNA-21-r	GCGGTTTTAGGGGATTGTAACCCCGCAGAGTCCCGCAAACTCTTTATTTTA GTCCCTTTTCAGGGACTAAAACGCCAAGCGCACCTAATTTCCCTATAGTG AGTCGTATTAGGATCC
sgRNA-22-r	GCGGTTTTAGGGGATTGTAACCCCGCAGAGTCCCGCAAACTCTTTATTTTA GTCCCTTTTCAGGGACTAAAACGCCAAGCGCACCTAATTTCCCCTATAGT GAGTCGTATTAGGATCC
sgRNA-23-r	GCGGTTTTAGGGGATTGTAACCCCGCAGAGTCCCGCAAACTCTTTATTTTA GTCCCTTTTCAGGGACTAAAACGCCAAGCGCACCTAATTTCCCCCTATAG TGAGTCGTATTAGGATCC
L58Y-f	TACGCTCGGCGGAAGGCTCGCCTCAACC
L58Y-r	CCTTTTTCGGGCGGAGCGGGCCAAC
D900K-f	AAAATGCAGGAACCAGAATTTGTTTATTATAACGC
D900K-r	TTTTGTCTGGATCAGAATCAGGCTATCTTTGTAG
PAM discovery assay-f	TAACTTACGGAGTCGCTCTACGGCCTGCAGGTCGACTCTAGAGGA
PAM discovery assay-r	GGATGGGATTCTTTAGGTCCTGGTTGTAAAACGACGGCCAGTGAA

Supplementary Table 3. Plasmids used for *in vivo* editing experiments.

Name	Sequence
pSI-545-CjCas9	https://benchling.com/s/seq-zLEZh1m0yJrUq7cQ1gH6
pSI-545-enCjCas9	https://benchling.com/s/seq-jbvjF5FuyHa9PtL3yPGI
pSI-545-CjCas9-AID	https://benchling.com/s/seq-7GGPB5DNrlkrpxYitenWa
pSI-545-enCjCas9-AID	https://benchling.com/s/seq-aUoM8Pwzn6HJimXkjT8h
pSI-414-CjCas9-sgRNA	https://benchling.com/s/seq-PZZASBMREPo41CtpOhzZ

Supplementary Table 4. Target sequences for genome- and base-editing analyses.

Gene	Sequence	PAM
DYRK1A	GGGCAAATTTCAGGTAGAAGAA	N_3AACAC
EMX1	GCCCAGCATGCTGCCCTTGACT	N_3AACAC
MECP2	GACACCCTCCTGAAAGCTGTCT	N_3AACAC
DYRK1A	GGTTCTCCTATTCTCAGGTCCA	N_3GACAC
EMX1	GCATCAAAACAAAAGGGAGATT	N_3GACAC
MECP2	GCCCTCACCAGGGGCTGAATGT	N_3GACAC
PTEN	GTAACTTCTGACATGCAATATA	N_3GACAC
DYRK1A	GACCGGGTTATTTGTTGCCATG	N_3CACAC
EMX1	GCCCCACATGGGCAGACACATG	N_3CACAC
MECP2	GGACAAAATGATGAGGGTCAAG	N_3CACAC
PTEN	GGCCAAACTCCAGAGATAACTA	N_3CACAC
DYRK1A	GTTTGCTACACTCTGGTTTCTT	N_3AGCAC
EMX1	GAGTCACCCAGGTTGCCCACCT	N_3AGCAC
MECP2	GCTAGAAGTCCAAGATCAAGGT	N_3AGCAC
PTEN	GTTCCTGTTTCTCCACATCCTC	N_3AGCAC
DYRK1A	GCAGCCTCCTGTGTTGTTCTCA	N_3GGCAC
EMX1	GTGCAACATGTGTCTGCCCATG	N_3GGCAC
MECP2	GGCAGAAGAGACAGGGAAGGAG	N_3GGCAC
PTEN	GGCCTCCTCTTTGCTGATTCTA	N_3GGCAC
DYRK1A	GAGACACACAGTCCCCAGGTGA	N_3CGCAC
EMX1	GAGGCTGGGACCTGGACCTTGC	N_3CGCAC
MECP2	GGCCGGGATTACAGGCGTCAGC	N_3CGCAC
PTEN	GAAACGTTAAGGCCAGGCATGA	N_3CGCAC
DYRK1A	GACTCACTAGAAAGGGAAATGG	N_3TACAC
EMX1	GATCTCTCCTTTCTACAATTCG	N_3TACAC
MECP2	GCCAGAGTCACCTAGAGCCACT	N_3TACAC
PTEN	GGACCATATACCATCTCCAGCT	N_3TACAC
DYRK1A	GTCATGAGTCTCCTGGTTGTCT	N_3AACAA
EMX1	GTACTCAGATGCTGAGGAGCCT	N_3AACAA
MECP2	GCAATCTGTGGCTTAGTATTTG	N_3AACAA
PTEN	GCTCGGTAATCCGGTCTCCTAA	N_3AACAA
DYRK1A	GCTTCCCACACCAACCAGAATG	N_3AACAT
EMX1	GACCTCTGTGAGGAGTAAATGA	N_3AACAA
MECP2	GAAACGACTCTGGTTGGAAATT	N_3AACAA
DYRK1A	GCGTTTCAAATGCACTGAAAGA	N_3AACAG
EMX1	GCTCCGTATGATAAGTTATGAG	N_3AACAG

MECP2	GTCTTCATGTTCCCACTTCAAA	N_3AACAG
PTEN	GTTACTTTCCATGTATTGGATA	N_3AACAG
PTEN	GGGGGCTGGAAGGAATATTAGG	NGGAACAC
GRIN2B	GTGGAGCTAACAGTCTAGTTAG	NGGAACAC
EMX1	GGGAGGATTGGGGTCTGGGGGA	NGGAACAC
DHRS3	GAGGACAAAGTGGAATCTGGCA	NGGAACAC
TNFRSF1B	GTGTCCAGTAGCACAATTAGAT	NGGAACAC
MTOR	GTGTGTGGGGATATGGGGTCAG	NGGAACAC
CFAP74	GAGGAAGGGAGACTAAAGTGAC	NGGAACAC
CTNNBIP1	GAGGCAGTAGCTGCAAAGCCCT	NGGAACAC
CASZ1	GTGGAACCAGCTGCCTGCTCCA	NGGAACAC
CAMTA1	GTGTTTGGGGCAGTGTGATCAT	NGGAACAC
