# Supplementary material for: Engineered Campylobacter jejuni Cas9 variant with enhanced activity and broader targeting range
Source: Commun Biol. 2022 Mar 8;5:211. doi: 10.1038/s42003-022-03149-7 (PMC8904486; doi:10.1038/s42003-022-03149-7)
Supplement: Supplementary file 3 — Description of Additional Supplementary Files [file 42003_2022_3149_MOESM3_ESM.pdf]

Description of Additional Supplementary Files

File name: Supplementary Data 1
Description: Genome editing analyses in human cells.

File name: Supplementary Data 2
Description: Base editing analysis in human cells.

File name: Supplementary Data 3
Description: Specificities of CjCas9 and enCjCas9.

File name: Supplementary Data 4
Description: The source data for the graphs in the main figure.
